# Comparison of Phytochemical Composition, Antibacterial, and Antifungal Activities of Extracts from Three Organs of *Pistacia lentiscus* from Saudi Arabia

**DOI:** 10.3390/molecules28135156

**Published:** 2023-07-01

**Authors:** Maha Al-Zaben, Mayasar Al Zaban, Souheila Naghmouchi, Albandary Nasser Alsaloom, Nada Al-Sugiran, Ahlam Alrokban

**Affiliations:** 1 Chemistry Department, King Saud University, P.O. Box 11495, Riyadh 11452, Saudi Arabia; mzaben@ksu.edu.sa; 2 Biology Department, College of Science, Princess Nourah bint Abdulrahman University, Riyadh 11671, Saudi Arabia; analsaloom@pnu.edu.sa (A.N.A.); nialsugiran@pnu.edu.sa (N.A.-S.); ahalrokban@pnu.edu.sa (A.A.); 3 National Research Institute of Rural Engineering, Water, and Forestry, University of Tunis Carthage, Street of Hedi Karay BP.N°10, Ariana 2080, Tunisia; n.souheila@yahoo.com

**Keywords:** *Pistacia lentiscus*, HPLC analysis, phenolic compounds, antifungal activity, antibacterial activity

## Abstract

This in vitro study focused on the antimicrobial activity of methanolic and aqueous extracts of three organs (stems, roots, and leaves) of *Pistacia lentiscus* against nine bacterial species, two fungal, and one yeast strain. A comparative study of the yield, high-performance liquid chromatography (HPLC) composition, and polyphenol content of the different extracts was conducted. The obtained data showed that the yield of the methanolic extracts (between 13% and 33.3%) was greater than that of the aqueous extracts (between 10% and 18%). The highest yield recorded was in the presence of the methanolic leaf extract, followed by the stem and root extracts. Methanolic extracts are richer in polyphenols than aqueous extracts. Indeed, the highest content was observed in the leaf methanolic extract (28.4 mg GAE/g), followed by the stem (2.96 mg GAE/g), and then the root (2.62 mg GAE/g). HPLC revealed variability in the chemical compositions of the different methanolic extracts. The leaf extract was richer in phenolic acids and flavonoids than the stem and root extracts. Regarding antimicrobial activity, it was concluded that the different methanolic extracts of lentisk were more active than the aqueous extracts. In addition, the different methanolic extracts exhibited antimicrobial activity against all tested strains, despite their morphology and Gram-staining. Indeed, the maximum inhibition zones and the minimum inhibitory concentrations for the bacterial strains sensitive to the different methanolic extracts of the mastic tree were in the range of 7 to 11 mm and 12.5 to 25 µg/mL, respectively. In addition, these extracts showed greater inhibitory activity against the tested fungal strains (*A. niger* and *A. flavus*) and yeast (*C. albicans*) than against the bacterial strains. These extracts can be used to treat antimicrobial infections and as food preservatives.

## 1. Introduction

*Pistacia lentiscus* is known for its medicinal properties [1]. Decoction of the dried roots is effective against intestinal and stomach inflammation and in the treatment of ulcers [2]. The aerial part of *P. lentiscus* is widely used in traditional medicine to treat arterial hypertension because of its diuretic properties [3,4]. The leaves have anti-inflammatory, antibacterial, antifungal, antipyretic, astringent, hepatoprotective, expectorant, and stimulant action [5,6]. They are also used in the treatment of other diseases in different countries, such as Palestine [7], Israel [8], the Kingdom of Jordan [9], the Golan Heights, the West Bank region [10], Turkey [11,12], and Egypt [13].

The resin obtained from *P. lentiscus* is known for its analgesic, antibacterial, antifungal, antioxidant, antiatherogenic, expectorant, stimulatory, diuretic, and spasmolytic effects [14,15,16].

*P. lentiscus* resin has traditionally been considered an anticancer agent, particularly against breast, liver, stomach, spleen, and uterine tumors. These traditional beliefs are consistent with recent studies showing that Chios mastic induces apoptosis [17] and has an anti-proliferative action against colon cancer cells [18]. Lentisk essential oil is known for its therapeutic advantages in the treatment of lymphatic and circulatory conditions. Previous studies on the essential oils of *P. lentiscus* have revealed some biological activities. Ali-Sgtayeh et al. [19] and Duru et al. [20] showed that the *P. lentiscus* essential oils (EO) possess significant antifungal activity. Their anti-inflammatory and anti-phospholipase activities have been demonstrated by Giner-Larza et al. [21,22]. Gardeli et al. [23] demonstrated that the *P. lentiscus* EO exhibited the highest free radical scavenging activity and antioxidant capacity. Mehenni et al. [24] showed that *P. lentiscus* exhibits promising antidiabetic activity in streptozotocin-induced diabetic rats, similar to that of the reference drug glibenclamide.

The high-performance liquid chromatography (HPLC) composition of methanolic extracts of three organs (stems, fruits, and leaves) of mastics collected from Tunisia has been evaluated by Yemmen et al. [25]; however, no correlation between the antibacterial activity and the methanolic extract composition of mastic harvested from Saudi Arabia has been reported. We were interested in an in vitro study of the antimicrobial activity of the methanolic and aqueous extracts of three organs (stems, roots, and leaves) of *P. lentiscus* collected from Saudi Arabia against nine bacterial, two fungal, and one yeast strain, all of which are pathogenic. Thus, a comparative study of the yield, polyphenol content, HPLC composition, and antimicrobial activity of different extracts was conducted.

## 2. Results and Discussion

### 2.1. Yield of Methanolic and Aqueous Extraction

**Figure 1:**
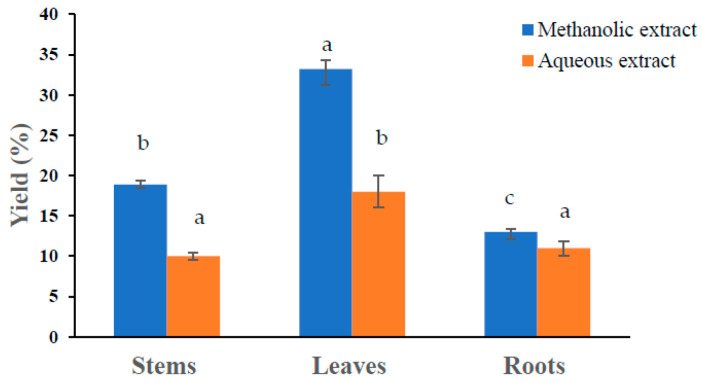
Yield (%) of methanolic and aqueous extracts of different organs of *P. lentiscus*. Values given are means (error bars represent standard deviations) of three independent experiments. Means with different letters are different (*p* < 0.05).

Calculating the yields not only makes it possible to assess the total extracts from the studied species, but also to consider the number of organs to be removed if necessary for a similar study, which would make the rational and sustainable use of the target species possible [26]. In this study, the yields of two extracts (aqueous and methanolic) from the stems, roots, and leaves of *P. lentiscus* were estimated. The comparison of the yields of different lentisk extracts showed that the yield of methanolic extracts (between 13% and 33.3%) was greater than that of aqueous extracts (between 10% and 18%) (Figure 1). This observation is consistent with the results of Yemmen et al. [25] and Zitouni et al. [27] and differs from that obtained by Spigno et al. [28]. These authors showed that aqueous solvents gave the best extraction yields compared to methanol, ethanol, and acetone [28]. In contrast, the highest yield was observed in the methanolic leaf extract, followed by the stem and root extracts. This observation is consistent with that of a previous study, which demonstrated that the highest amounts of phenolic compounds were observed in leaves [29,30]. Hudaib et al. [31] explained this as the existence of an interaction between organs and different biosynthesis, degradation, and transport processes involved in the distribution of these polyphenols at the plant level. The different returns found in this study are lower than those obtained in other studies. Furthermore, Atmani et al. [32] reported that the extraction yield from different organs of *P. lentiscus* was 44.58%. In general, the dry extract content varies according to the parameters of the extraction process, the size of the particles constituting the sample, time and storage conditions, and the presence of interfering substances [33]. The solvent is an essential parameter that can also affect the extraction of polyphenols [34].

### 2.2. Chemical Composition

**Figure 2:**
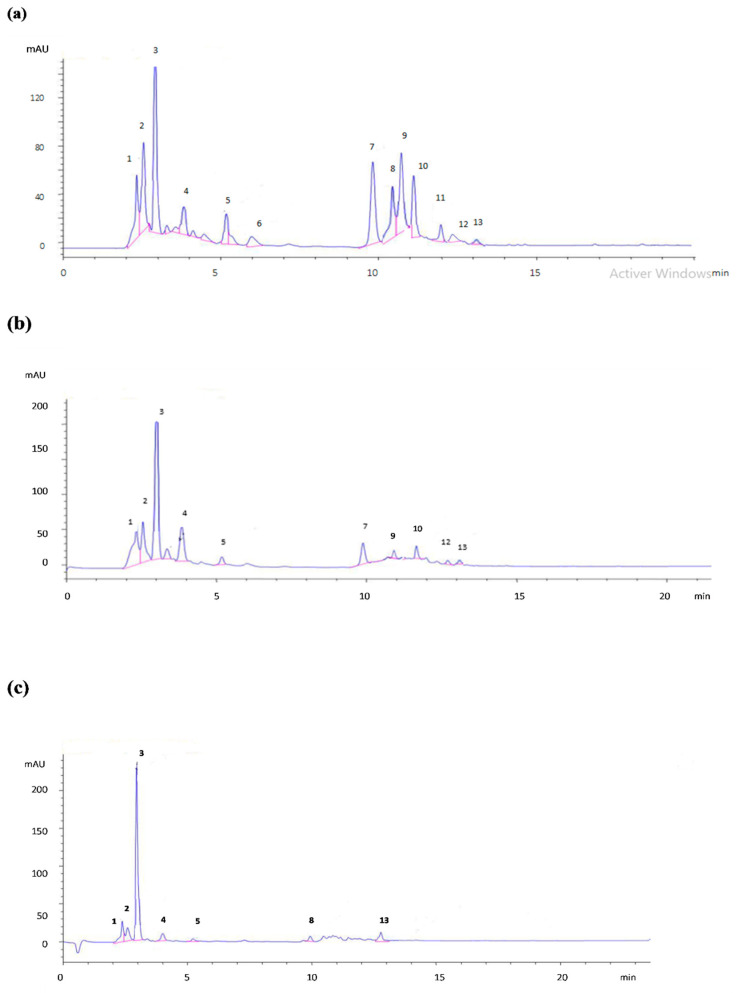
HPLC Chromatographic profiles of phenolic compounds, monitored at 280 nm, in the methanolic extract of *P. lentiscus* leaves (**a**), roots (**b**), and stems (**c**).

HPLC was used to determine the chemical composition of the methanolic extracts from the three organs of *P. lentiscus* (Figure 2). The evaluated extracts were compared to a mixture of standard compounds. Phenolic compounds were identified by comparing the congruent retention times with standards. The data summarized in Table 1 show the presence of significant qualitative and quantitative differences in the chemical composition of the extracts from different *P. lentiscus* organs. The leaf extract was richer in phenolic compounds than the root and stem extracts. The leaf extract contained 13 compounds (tannic acid, gallic acid, digalloyl quinic acid derivative, chlorogenic, cafeic, vanillic, p-coumaric, ferulic acid, catechin hydrate, epicatechin, taxifolin, quercetin, and myricetin). In addition, ten (tannic acid, gallic acid, digalloyl quinic acid derivative, chlorogenic, cafeic, vanillic acid, catechin hydrate, taxifolin, quercetin, and myricetin) and seven (tannic acid, gallic acid, digalloyl quinic acid derivative, chlorogenic acid, catechin hydrate, p-coumaric acid, and myricetin) phenolic compounds were identified in the stem and root methanolic extracts, respectively. Our results are comparable to those of a previous study on *P. lentiscus* collected from Tunisia [25]. According to previous studies, the leaf extract of *P. lentiscus* seems to be richer in phenolic compounds than fruit and stem extracts, and 14 phenolic compounds have been identified, including eight phenolic acids (tannic acid, gallic acid, 3,5-O-digalloyl quinic acid, chlorogenic, cafeic, vanillic, p-coumaric, ferulic, 3,4-dihydroxybenzoic, and trans-cinnamic acid) and six flavonoids (catechin hydrate, epicatechin, taxifolin, quercetin, and myricetin). Azaizeh et al. [35] signaled the presence of gallic acid, catechin, digalloyl quinic acid, chlorogenic acid, and rutin in *P. lentiscus* collected from Israel.

### 2.3. Secondary Metabolite Content

**Figure 3:**
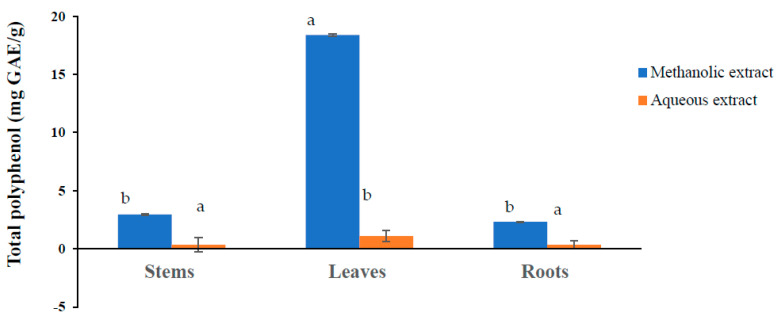
Total phenolic content in methanolic and aqueous extracts of different lentisk organs. Values given are means (error bars represent standard deviations) of three independent experiments. Means with different letters are different (*p* < 0.05).

Total phenols were determined by the spectrophotometric method using the Folin–Ciocalteu reagent. For the two extracts prepared from the three organs of *P. lentiscus*, variability in the total phenol content was observed (Figure 3). Methanolic extracts are richer in polyphenols than aqueous extracts. Yemmen et al. [25] explained the richness of the methanolic extract by phenolic compounds compared to other solvents due to their polarities and good solubility.

In addition, the highest content was observed in the leaf methanolic extract, followed by the stem and root extracts; the results were 28.4, 2.96, and 3.62 mg GAE/g, respectively. These essential results reflect the data shown in Figure 2, in which we recorded high yields of methanolic extracts, especially from the leaves, demonstrating the richness of this part of the plant in polyphenols. The polyphenol content of various lentisk extracts was higher than those reported by Gardeli et al. [23]. Indeed, these authors showed that the phenolic content of this plant was approximately 0.588 g of gallic acid/g. Similarly, Zitouni et al. [27] showed that the total phenolic content in the methanolic extracts of different organs of *P. lentiscus* varied between 216.289 ± 20.62 mg GAE/g DM (leaves) and 103.342 ± 2.317 mg/g (fruits). However, further studies are needed because the Folin–Ciocalteu assay does not distinguish between different phenolic compounds. Substances such as sugars, aromatic amines, ascorbic acid, sulfur dioxide, and iron can interfere with the Folin–Ciocalteu assay, and corrections for interfering substances must be made to accurately measure the phenolic content of the samples [36]. Inorganic substances can also interact with the Folin–Ciocalteu reagent, which can lead to erroneous results. Another parameter that must be considered when applying the Folin–Ciocalteu assay is the structural characteristics of phenolic compounds [37]. The molar response of this method is roughly proportional to the number of structural differences reported to be responsible for antioxidant activity.

In general, plant materials rich in phenolic compounds are increasingly used in the food industry because they retard the oxidative degradation of lipids and improve the quality and nutritional value of foods [38]. Phenolic compounds are considered secondary metabolites. Phytochemicals derived from phenylalanine and tyrosine are ubiquitous and diverse [34]. The high levels of total phenolic compounds in the methanolic lentisk extracts may contribute to their significant antifungal and antimicrobial activities. These results justify the use of the different plant organs by the local population for the treatment of certain diseases.

### 2.4. Antimicrobial Activities of Methanolic and Aqueous Extracts

Faced with the problem of microbial resistance to synthetic antibiotics, much work has been conducted on the antimicrobial activity of natural products extracted from plants. In this study, we tested the antimicrobial potency of the methanolic and aqueous extracts of *P. lentiscus* against nine pathogenic strains that cause foodborne diseases, two fungal, and one yeast strain. This power was evaluated by determining the diameter of the inhibition zones (Table 2), MIC and MCB concentrations (Table 3).

From the analysis of the results obtained during the study of antimicrobial activity, it can be concluded that the various methanolic extracts of the mastic tree are more active than the aqueous extracts. This variability may be due to differences in the polyphenol contents of the two types of extracts tested. The antimicrobial effects of phenols have long been known. It has been reported that the antimicrobial activities of plant extracts depend on the nature and structure of phenolic compounds. Through their hydroxyl groups and phenolic compounds, they can bind to proteins in bacterial membranes to form complexes [39]. Several studies have demonstrated the antimicrobial activity of plant extracts from various organs, such as leaves, seeds, and flowers [40,41]. Plant antimicrobial compounds inhibit bacterial growth via various mechanisms. Caillet and Lacroix [42] showed that the antimicrobial action of essential oils and their extracts occurs in three phases: (i) attack of the bacterial wall by the extracts, causing an increase in permeability and subsequent loss of cellular constituents; (ii) acidification of the interior of the cell, blocking the production of cellular energy and the synthesis of structural components; and (iii) destruction of genetic material, leading to bacterial death. Therefore, antimicrobials may have relevant clinical value in the treatment of resistant microbial strains. Sarac and Ugur [43] revealed that vegetable oils and their extracts are used in several products, including food, cosmetics, and pharmaceuticals, as well as in alternative medicine and therapies.

The maximum inhibition zone and the values of the MIC for the bacterial strains sensitive to the different methanolic extracts of lentisk were in the range of 7 to 11 mm and 1.25 to 2.5 µg/mL, respectively. The minimum bactericidal concentrations vary between 2.5 and 5 µg/mL. These extracts exhibited antimicrobial activity against Gram-positive (*B. cereus*, *L. monocytogens*, *S. arizona*, *S. aureus*) and Gram-negative bacteria (*E. coli*, *P. aeruginosa*, *S. typhimurium*, *K. pneumoniae*). In addition, the obtained data show that the different methanolic extracts (MIC varied between 1.25 to 2.5 µg/mL) have a greater effect than standard antibiotics (MIC between 1 and 4 µg/mL) against all tested strains.

Previous studies have shown that the majority of essential oils and their extracts tested for antimicrobial properties have a more pronounced effect against Gram-positive bacteria. The resistance of Gram-negative bacteria is attributed to their hydrophilic outer membrane, which blocks the penetration of hydrophobic compounds into the target cell membrane [44,45]. In this study, the different methanolic extracts of lentisk exhibited antimicrobial activity against all tested strains, despite their morphology and Gram staining. This observation is consistent with previous studies carried out on several medicinal plants [46,47].

The antifungal activities summarized in Table 4 and Table 5 show that the various methanolic extracts of lentisk have inhibitory activities against fungal strains (*A. niger* and *A. flavus*) and yeast (*C. albicans*). The diameters of the inhibition zones and the MIC values varied between 8 and 11 mm and 1.56 and 25 µg/mL, respectively. In addition, this activity is essential compared to that observed against other bacterial strains. This result agrees with those reported by Iauk et al. [48] and Ali-Shtayeh and Abu Ghdeib [19]. These authors reported that the methanolic extracts of lentisk have greater antifungal than antibacterial activity. By analyzing the results of the antimicrobial and antifungal activities, we can conclude that bacteria that showed significant inhibition zones obtained by the method of diffusion on agar medium do not always generate higher MIC and MBC values. Based on these results, it can be concluded that the volatility of the extract affects the antimicrobial activity as determined by the disc diffusion method. Similarly, previous studies have reported that strains that exhibit large inhibition zones are not always the most sensitive (MIC and MFC values are lower) because the diameters of the inhibition zone do not reflect the antibacterial efficacy of the tested product. This can be attributed to the solubility and evaporation of the extract [49,50].

## 3. Materials and Methods

### 3.1. Plant Material and Yield Determination

The current study was carried out on the leaves, roots, and stems of *P. lentiscus* harvested in the agronomical region situated in Al-Kharj in Saudi Arabia and identified according to the ”Flora of The kingdom of Saudi” by Chaudhary et al. [51]. The different parts were ground into a fine powder after shade drying. The voucher specimens of used plants were AQJ_61.

### 3.2. Preparation of Extracts

To prepare aqueous extracts, 80 g of each plant material was macerated in 800 mL of distilled water for 48 h. The solvents were then evaporated in an oven at 65 °C. The dry extracts were collected, weighed, and suspended in 50 mL of distilled water.

The methanolic extracts were prepared by macerating 10 g of the powdered plant material in 100 mL methanol for 24 h. The solution was filtered and kept at room temperature until the solvent completely evaporated. Dry extracts were collected, weighed, and suspended in 50 mL of methanol.

The yield, expressed as a percentage (%) of each extract, was determined relative to the mass of the material using the following formula:

R (%) = M/M0

where M is the mass of the resulting dry extract (mg) and M0 is the mass of the plant material to be treated (g).

### 3.3. HPLC Analysis

HPLC analysis of different extracts was performed as described by Al. Zaban et al. [52]. An Agilent 1100 series HPLC system was used to separate the phenolic compounds. The system was equipped with an inline degasser (G 1322A), a quaternary pump (G 1311A), thermostatic automatic sampler (G 1313A), column heater (G 1316A), and diode array detector (G 1315A). In addition, Agilent HPLC Chemstation 10.1 edition under Windows 2000 was used to control and analyze the data. Separation was performed using an ODS C18 column in the reverse phase (4 mm, 250 mm × 4.6 mm), which was used as the stationary phase at room temperature. Acetonitrile (solvent A) and water with acetic acid (solvent B (0.2 mL/100 mL water)) were used as the mobile phases. The gradient program was as follows: 15% A/85% B 0–12 min, 40% A/60% B 12–14 min, 60% A/40% B 14–18 min, 80% A/20% B 18–20 min, 90% A/10% B 20–24 min, and 100% A 24–28 min. The flow rate was maintained at 0.5 mL/min. The injection volume was 20 μL and the peaks were monitored at 280 nm. Peaks were identified by comparing the retention time and UV spectra of the phenolic chromatogram of the fractions with those of pure standards purchased from Sigma-Aldrich (St. Louis, MO, USA). Preparation of stock solutions (1 μg/mL) of different standards was carried out by dissolving 5 mg of the compound in 5 mL of HPLC-grade methanol. The solutions were then stored at −20 °C.

### 3.4. Determination of Secondary Metabolite Content

The phenolic content in extracts prepared from *P. lentiscus* was estimated using the method previously reported by Slinkard and Singleton [53]. Folin–Ciocalteu reagent and gallic acid were obtained from Sigma-Aldrich. A gallic acid stock solution (0.5 g/L) was prepared using distilled water.

The prepared extract (50 µL) was mixed with 3.95 mL water and 25 µL Folin–Ciocalteu reagent. The 20% aqueous solution of sodium carbonate was added after 5 min at a volume of 750 µL. The volume was adjusted to 5.0 mL using distilled water. The resulting mixture was vortexed and maintained in a water bath for 30 min at 40 °C. After 5 min at ambient temperature, the absorbance was measured at 765 nm. Gallic acid was used as the standard and calibration curves were constructed by measuring the absorption of gallic acid solutions of different concentrations (0.0125–0.5 mg/mL).

The obtained data were expressed as milligram gallic acid equivalents per gram dry weight (mg GAE/g DW) using the linear regression equation of the calibration curve (R^2^ = 0.971) plotted for gallic acid.

### 3.5. Evaluation of Antimicrobial Activity

In the current study, the antimicrobial activities of different extracts of *P. lentiscus* were evaluated against nine bacterial and three fungal strains using two different methods. The different strains and culture conditions are summarized in Table 6. Qualitative and quantitative antimicrobial evaluations were performed using two methods: disc diffusion assay and broth dilution method.

The antimicrobial activities of the extracts prepared from *P. lentiscus* were quantitatively evaluated using a disc diffusion assay [54]. Furthermore, 100 µL of suspension containing 10^8^ CFU/mL of bacterial cells, 10^6^ CFU/mL of yeast, and 10^4^ spores/mL of fungi were spread on petri plates containing TSA, SDA, and PDA, respectively. Sterile filter paper discs (6 mm in diameter) were separately impregnated with 15 μL of tested extract and placed on the agar which had previously been inoculated with the selected bacteria. Gentamicin (10 µg/disc) and amphotericin B (20 µg/disc) were used as positive reference for bacteria and fungi, respectively. A disc without samples served as the negative control. The solvent activity was determined using a control disc soaked in the extract. The inoculated plates were incubated under the appropriate conditions. The qualitative antimicrobial activity of the tested extract was estimated by measuring the diameter of the growth inhibition zone (including the disc diameter of 6 mm).

The broth dilution method was used to estimate the minimum inhibitory concentrations (MIC) and minimum bactericidal concentrations (MBC) of the prepared extracts as described by Aouadhi et al. [54]. The presence of turbidity and a “pellet” on the tube bottom was used as an indicator of microbial growth. The lowest concentration in each row that completely inhibited the bacterial growth corresponded to the MIC. The MBC is usually an extension of the MIC, where the microorganisms quantitatively indicate the minimum concentration and no viable organism appears in the culture [54].

### 3.6. Statistical Analysis

All experiments were repeated thrice, and the results were presented as means ± standard deviation. Analysis of variance was performed using SPSS 14.0 for Windows (IBM Corp., SPSS Inc., Armonk, NY, USA). Significant differences between the means were determined using Tukey’s post hoc tests. Differences were considered statistically significant at *p* < 0.05.

## 4. Conclusions

In conclusion, the data obtained in the current investigation highlight the presence of significant variations in polyphenol composition and antimicrobial activities among the organs and types of extracts prepared from *P. lentiscus* harvested in Saudi Arabia. The methanolic extracts are richer in polyphenols than aqueous extracts. The highest content was observed in the methanolic leaf extract. In addition, HPLC analysis demonstrated that the leaf extract was richer in phenolic acids and flavonoids than the stem and root extracts. Moreover, these extracts exhibited strong antiradical and antibacterial activities. Based on these data, the methanolic extract of *P. lentiscus* could be used as an antimicrobial agent to treat infectious diseases in humans.

## Figures and Tables

**Table 1:** Chemical identification by HPLC of methanolic extracts of three organs of *P. lentiscus*.

Retention Time	Compounds	Leaves (%)	Roots (%)	Stems (%)
2.3	Tannic acid (1)	9.1 ± 0.00 ^b^	6 ± 0.07 ^c^	12.0 ± 0.04 ^a^
2.5	Gallic acid (2)	12 ± 0.03 ^a^	8.2 ± 0.06 ^b^	8.31 ± 0.05 ^b^
2.9	3,5-*O*-digalloyl quinic acid	41.11 ± 0.05 ^b^	23 ± 0.09 ^c^	52 ± 0.09 ^a^
3.8	Catechin hydrate (4)	9.45 ± 0.02 ^a^	3.8 ± 0.02 ^b^	1.92 ± 0.03 ^b^
5.2	Chlorogenic acid (5)	5.11 ± 0.06 ^a^	1.07 ± 0.02 ^b^	0.7 ± 0.01 ^b^
6.37	Epicatechin (6)	1.36 ± 0.07 ^a^	Nd	Nd
9.95	Vanillic acid (7)	12.5 ± 0.03 ^a^	1.77 ± 0.05 ^b^	Nd
10.1	p-coumaric acid (8)	9.63 ± 0.03 ^a^	Nd	0.61 ± 0.01 ^b^
10.5	Taxifolin (9)	16.01 ± 0.02 ^a^	0.43 ± 0.04 ^b^	Nd
10.8	Quercetin (10)	11 ± 0.0 ^a^	0.45 ± 0.05 ^b^	Nd
12.1	Ferulic acid (11)	4.46 ± 0.00 ^a^	Nd	Nd
12.02	Rutin hydrate (12)	2.2 ± 0.02 ^a^	0.65 ± 0.01 ^b^	Nd
13.09	Myricetin (13)	0.6 ± 0.00 ^a^	0.5 ± 0.03 ^a^	1.2 ± 0.02 ^b^

Results are expressed as mean ±  standard deviation of three determinations. Different letters in the same line indicate significant differences (*p* < 0.05). Nd: not determined.

**Table 2:** Diameters of the inhibition zones obtained by the effect of different lentisk extracts against nine bacterial species.

Bacterial Strains	Diameters of the Inhibition Zones (mm)							
	Aqueous Extracts			Methanolic Extracts			Antibiotic (Gentamicin)	Solvent Extract
	Stems	Leaves	Roots	Stems	Leaves	Roots		
*E. coli*	-	-	-	7 ± 0.5 ^b^	8 ± 0.4 ^a^	7 ± 0.5 ^a^	24 ± 1.1 ^c^	-
*S. arizona*	-	-	-	8 ± 0.6 ^a^	7 ± 0.6 ^a^	7 ± 0.7 ^a^	23 ± 0.8 ^c^	-
*S. typhimurium*	-	-	-	8 ± 0.9 ^a^	8 ± 0.5 ^a^	8 ± 0.6 ^a^	21 ± 0.9 ^b^	-
*L. monocytogenes*	-	-	-	9 ± 0.6 ^b^	10 ± 0.3 ^b^	8 ± 0.4 ^a^	17 ± 1 ^a^	-
*K. pneumoniae*	-	-	-	10 ± 0.7 ^b^	11 ± 0.2 ^c^	9 ± 0.3 ^b^	18 ± 0.7 ^a^	-
*S. aureus*	-	-	-	10 ± 0.6 ^b^	10 ± 0.6 ^b^	9 ± 0.6 ^b^	21 ± 1.2 ^b^	-
*B. subtillis*	-	-	-	9 ± 0.8 ^b^	11 ± 0.5 ^c^	9 ± 0.4 ^b^	16 ± 0.9 ^a^	-
*A. hydrophila*	-	-	-	8 ± 0.5 ^a^	9 ± 0.7 ^b^	8 ± 0.6 ^a^	22 ± 0.8 ^b^	-
*P. aeruginosa*	-	-	-	8 ± 0.7 ^a^	8 ± 0.6 ^a^	7 ± 0.3 ^a^	21 ± 0.5 ^b^	-

-: Not active. Values given are means (error bars represent standard deviations) of three independent experiments. Means with different letters are different (*p* < 0.05).

**Table 3:** Minimum inhibitory and minimum bactericidal concentrations of different *P. lentiscus* extracts against nine bacterial species.

Microbial Strains	Methanolic Extracts							
	Minimum Inhibitory Concentrations (µg/mL)				Minimum Bactericidal Concentration (µg/mL)			
	Stems	Leaves	Roots	Antibiotics	Stems	Leaves	Roots	Antibiotics
*E. coli*	2.5 ± 0.0 ^a^	2.5 ± 0.0 ^a^	2.5 ± 0.0 ^a^	4 ^c^	5 ± 0.0 ^a^	5 ± 0.0 ^a^	5 ± 0.0 ^a^	8 ± 0.0 ^c^
*S. arizona*	2.5 ± 0.0 ^a^	2.5 ± 0.0 ^a^	2.5 ± 0.0 ^a^	4 ^c^	5 ± 0.0 ^a^	5 ± 0.0 ^a^	5 ± 0.0 ^a^	8 ± 0.0 ^c^
*S. typhimurium*	2.5 ± 0.0 a	2.5 ± 0.0 ^a^	2.5 ± 0.0 ^a^	4 ^c^	5 ± 0.0 ^a^	5 ± 0.0 ^a^	5 ± 0.0 ^a^	8 ± 0.0 ^c^
*L. monocytogenes*	1.25 ± 0.0 ^b^	1.25 ± 0.0 ^b^	2.5 ± 0.0 ^a^	2 ^b^	2.5 ± 0.0 ^b^	5 ± 0.0 ^a^	5 ± 0.0 ^a^	4 ± 0.0 ^b^
*K. pneumoniae*	1.25 ± 0.0 ^b^	2.5 ± 0.0 ^a^	2.5 ± 0.0 ^a^	1 ^a^	2.5 ± 0.0 ^b^	5 ± 0.0 ^a^	5 ± 0.0 ^a^	2 ± 0.0 ^a^
*S. aureus*	2.5 ± 0.0 ^a^	2.5 ± 0.0 ^a^	2.5 ± 0.0 ^a^	1 ^a^	5 ± 0.0 ^b^	5 ± 0.0 ^a^	5 ± 0.0 ^a^	2 ± 0.0 ^a^
*B. subtillis*	1.25 ± 0.0 ^b^	2.5 ± 0.0 ^a^	2.5 ± 0.0 ^a^	2 ^b^	2.5 ± 0.0 ^b^	5 ± 0.0 ^a^	5 ± 0.0 ^a^	4 ± 0.0 ^b^
*A. hydrophila*	2.5 ± 0.0 ^a^	2.5 ± 0.0 ^a^	2.5 ± 0.0 ^a^	2 ^b^	5 ± 0.0 ^a^	5 ± 0.0 ^a^	5 ± 0.0 ^a^	4 ± 0.0 ^b^
*P. aeruginosa*	2.5 ± 0.0 ^a^	2.5 ± 0.0 ^a^	2.5 ± 0.0 ^a^	2 ^b^	5 ± 0.0 ^a^	5 ± 0.0 ^a^	5 ± 0.0 ^a^	4 ± 0.0 ^b^

Values given are means (error bars represent standard deviations) of three independent experiments. Means with different letters are different (*p* < 0.05).

**Table 4:** Diameters of the inhibition zones obtained by the effect of different lentisk extracts on two fungi and one yeast.

*C. albicans*	-	-	-	10 ± 0.4 ^c^	11 ± 0.5 ^c^	10 ± 0.2 ^b^	20 ± 1 ^c^	-

-: Not active. Values given are means (error bars represent standard deviations) of three independent experiments. Means with different letters are different (*p* < 0.05).

**Table 5:** Minimum inhibitory and fungicidal concentrations of different lentisk extracts against two fungi and one yeast.

Strains	Minimum Inhibitory Concentration (µg/mL)			Minimum Fungicidal Concentration (µg/mL)		
	Stems	Leaves	Roots	Stems	Leaves	Roots
*A. niger*	12.5 ± 0.0 ^a^	12.5 ± 0.0 ^a^	25 ± 0.0 ^a^	25 ± 0.0 ^a^	25 ± 0.0 ^a^	50 ± 0.0 ^a^
*A. flavus*	3.12 ± 0.0 ^b^	3.12 ± 0.0 ^b^	1.56 ± 0.0 ^b^	6.25 ± 0.0 ^b^	6.25 ± 0.0 ^b^	3.12 ± 0.0 ^b^
*C. albicans*	3.12 ± 0.0 ^b^	3.12 ± 0.0 ^b^	1.56 ± 0.0 ^b^	6.25 ± 0.0 ^b^	6.25 ± 0.0 ^b^	3.12 ± 0.0 ^b^

Values given are means (error bars represent standard deviations) of three independent experiments. Means with different letters are different (*p* < 0.05).

**Table 6:** Different strains used to study the antimicrobial activity of various extracts and their culture conditions.

Microbial Groups	Strains	Growing Conditions
Gram-positive bacteria	*Listeria monocytogenes* ATCC 7644 *Bacillus subtillis* ATCC 6059 *Staphylococcus aureus* ATCC25923	Culture medium: trypto-casein agar soy (TCS). Incubation temperature: 37 °C.
Gram-negative bacteria	*Salmonella typhimurium* NCTC 6017 *Salmonella arizona* ATCC25922 *Pseudomonas aeruginosa* ATCC9027 *Escherichia coli* ATCC8739 *Aeromonas hydrophila* *Klebsiella pneumoniae*	
Yeast	*Candida albicans* ATCC 2091	Culture medium: Sabouraud Dextrose Agar (SDA). Incubation temperature: 30 °C.
Fungal species	*Aspergillus niger* *Aspergillus flavus*	Culture medium: Potate Dextrose Agar (PDA). Incubation temperature: 30 °C.

## Data Availability

Not applicable.
